# The Importance of ncRNAs as Epigenetic Mechanisms in Phenotypic Variation and Organic Evolution

**DOI:** 10.3389/fmicb.2017.02483

**Published:** 2017-12-22

**Authors:** Daniel Frías-Lasserre, Cristian A. Villagra

**Affiliations:** Instituto de Entomología, Universidad Metropolitana de Ciencias de la Educación, Santiago, Chile

**Keywords:** non-codingRNAs, phenotypic plasticity, biodiversity, adaptation, evolution

## Abstract

Neo-Darwinian explanations of organic evolution have settled on mutation as the principal factor in producing evolutionary novelty. Mechanistic characterizations have been also biased by the classic dogma of molecular biology, where only proteins regulate gene expression. This together with the rearrangement of genetic information, in terms of genes and chromosomes, was considered the cornerstone of evolution at the level of natural populations. This predominant view excluded both alternative explanations and phenomenologies that did not fit its paradigm. With the discovery of non-coding RNAs (ncRNAs) and their role in the control of genetic expression, new mechanisms arose providing heuristic power to complementary explanations to evolutionary processes overwhelmed by mainstream genocentric views. Viruses, epimutation, paramutation, splicing, and RNA editing have been revealed as paramount functions in genetic variations, phenotypic plasticity, and diversity. This article discusses how current epigenetic advances on ncRNAs have changed the vision of the mechanisms that generate variation, how organism-environment interaction can no longer be underestimated as a driver of organic evolution, and how it is now part of the transgenerational inheritance and evolution of species.

## Introduction

In the Synthetic Theory of Evolution, mutations have been proposed as the principal factor behind the origin of new phenotypic variation and highlighted as the cornerstone of evolutionary process (Nei, 2013). In that approach, phenotype variations related to the environment, such as the reaction norm and phenotypic plasticity, did not influence the genetic background, and were therefore not transmitted to offspring (Mayr, 1985). The central dogma postulates an unidirectional flow of information from DNA, mediated by RNA, to proteins (Crick, 1958, 1970). This pervasive idea consolidated a deterministic and reductionist inheritance (Shapiro, 2009; Frías-Lasserre, 2012), impacting our understanding of all genetic mechanisms that effectively intervene on population genetics and organic evolution (Schreiber, 2005; Weber, 2006; Gillings and Westoby, 2014). As a result, many evolutionary mechanisms have been omitted in Neo-Darwinian theory, including non-coding RNAs (ncRNAs; Frías, 2010). In classic evolutionary theory, genetic code was mainly associated with protein coding DNAs, which only make up ~2% of the human genome; however, recently, novel functions have been assigned for non-coding DNA regions for proteins (Lunter et al., 2006; Dunham et al., 2012). The remaining non-coding area of DNA has been revealed to be related to key biological processes and adaptive complexity in eukaryotic life, both in plants and animals, contradicting the paradox of the value C (Creevey and McInerney, 2003; Andolfatto, 2005; Taft et al., 2007; Knowles and McLysaght, 2009; Ling and Wurtele, 2014; Gaiti et al., 2016). In the habitat where the organisms live, there are a variety of stimuli and stressors that could induce rapid modification in the transcription of genes through epigenetic mechanisms capable of generating memory and epigenomic transgenerational inheritance (D'Urso and Brickner, 2014). Gene expression can be differentially influenced by stable epigenetic modifications that can be later kept through ontogenetic development with the aid of ncRNAs and even pass to the following generations (Jaenisch and Bird, 2003; Hanson and Skinner, 2016; Van Otterdijk and Michels, 2016).

The objective of this article is to analyze the importance of ncRNAs in the regulation of gene expression, and their impact at the level of population variability, adaptation, and the evolution of species. Also, we review and discuss how environmental stimuli and ncRNAs may play an important role in inheritance through the epigenome by triggering epigenetically heritable changes that may lead the origin of new species. In transgenerational inheritance caused by environmental stressors, ncRNAs may play an important role among the set of mechanisms that underlie changes in phenotypic variation and organic evolution.

## New mechanisms of genetic variability and phenotypic novelty

The stability of genes on homologous chromosomes, except for translocation, was a generalized fact for geneticists until 1960, when *mobile genetic elements* were described by Barbara McClintock (1950) in corn. This findings was later verified in other eukaryotes and prokaryotes (Sakaguchi, 1990; Kazazian, 2004). In addition to transposable elements, there are other epigenetic mechanisms explaining allelic instability and phenotypic variation such as: splicing, RNA editing, metastable epialleles, epimutation, and paramutations (Tollefsbol, 2014). Many of these mechanisms involve different ncRNAs capable of making gene regulation in cells of various tissues oriented to a wide range of biological processes (Yan, 2014).

## The epigenetic concept

Waddington (2012) coined *epigenetics* as *the interaction between genes and their products that allow for phenotypic expression* in order to reveal the mechanisms of development under the classic theory of epigenesis. Waddington also coined the concept of *epigenotype* as these *interphase that connected the genotype with the phenotype during development* (Slack, 2002; Sweatt and Tamminga, 2016). Epigenetics is a heritable change in the epigenotype of cells unchanged in the primary structure of DNA (Tollefsbol, 2011). Epigenetic was, for many years, limited to the understanding of cell differentiation. Now it is known that epigenetics is a hereditary transgenerational mechanism, linked to processes such as paramutations, metastable epialleles, DNA methylation, and chromatin remodeling, wherein there is also participation from different types of ncRNAs (Brink et al., 1968; Grewal and Klar, 1996; Cavalli and Paro, 1999; Kosten and Nielsen, 2014; Mashoodh and Champagne, 2014).

## NcRNAs

A major surprise arising from the DNA sequencing of eukaryotes organisms was the limited number of protein-coding genes found in relation to the total size of the genome. This had no correlation with the complexity of organisms, and did not explain the effects of selection pressure during evolution (Lander et al., 2001). In areas of the genome that do not encode for proteins, there is a great deal of information for ncRNAs, which also play a key role in regulating gene expression, working on specific sequences targeting genes, transposons, and viruses where they exert regulation or silencing (Mattick, 2009; Qu and Adelson, 2012). The first small RNAs were those rRNAs and tRNAs that were related to protein synthesis (Choudhuri, 2010). Currently, we know that there are many other classes of ncRNAs, small and long (Eddy, 2001), and know about their biogenesis, function and role in diseases (Choudhuri, 2010; Yu et al., 2014; Li et al., 2015). The most ancient of these small ncRNA is thought to be the ribozyme, which is a catalytic RNA. A ribozyme performs its catalyzing process without the aid of protein factors (Swati, 2017). The hammerhead ribozyme, 50–150 nt, was discovered in subviral plant pathogens and has been found in bacteria, archaea, and in many eukaryotic genomes, such as plants and mammals including the human genome. Some ribozymes, the riboswitches, have the ability to catalyze reactions in the absence of proteins and the capacity to function as switches that regulate gene expression by altering their conformation in response to a ligand or a small molecule. Some riboswitches act as thermosensors, detecting and alerting the organism of a temperature rise due to an infection or climatic change (Przybilski et al., 2005; Serganov and Dinshaw, 2007; Martick et al., 2008; De la Peña and Garcia-Robles, 2010a,b; Seehafer et al., 2011). The first ncRNA (miRNAs) was described in *Caenorhabditis elegans* and associated with embryonic development (Lee et al., 1993). In eukaryotes, they are relatively more abundant than protein-coding RNA (Herbert and Rich, 1999). For instance in humans, ~98% of all transcriptional output corresponds to ncRNAs, and was previously considered *junk DNA* (Wright and Bruford, 2011). NcRNAs have been detected in viruses, archaea, bacteria, and eucharia and can participate in a great number of cellular activities such as transcription, DNA replication, messenger RNAs stability, RNAs processing (Storz, 2002). There are different types of ncRNAs with varied functions; among the most relevant are:

### Micro RNA (miRNA)

Are short (22 bp), and found in animals, plants, and viruses. MiRNAs belongs to a highly conserved post-transcriptional regulatory gene family, with paramount functions across various cellular and developmental processes such as immunity, cell behavior (including proliferation, differentiation, contractility, inflammation), and host–microorganism interactions (Asgari, 2011; Mendell and Olson, 2012). In insects, miRNAs encoded by viruses interact with the host's defenses and help during virus replication (Asgari, 2013). In eutherian mammals, including humans, miRNAs from trophoblasts are expressed in the placentae of pregnant females and could mediate cross talk between the feto-placental unit and the mother during pregnancy (Ouyang et al., 2013). MiRNAs regulate several cellular processes in relation to pregnancy, such as: placental development, endometrial receptivity, angiogenes, and immune cells at the maternal-fetal interface. MiRNAs, are capable of regulating the immunological balance between the mother and her offspring, and likely help to regulate successful placentation and pregnancy. Also miRNAs, via exosomes, induce viral resistence through autophagy and has a role in the maternal-fetal exchange (Bidarimath et al., 2014). Furthermore, during pregnancy, miRNAs interact with reproductive hormones and are important regulators of mRNA translation (Bidarimath et al., 2014). The miRNAs resolve the paradoxical nature of mammalian pregnancy, in which an intimate immunological relationship exists between the mother and the allogeneic fetus where the mother does not reject the fetus. MiRNAs are packaged in vesicles within cells (nano-packages) and are released to the extracellular space, and circulate in blood and breast milk. These miRNAs carry out on target mRNAs in other distant or nearby cells, providing intercellular communication (Ouyang et al., 2013) and also induce antiviral immunity (Mouillet et al., 2014). In plants, miRNA may also play a critical role in seed development and germination (Pluskota et al., 2011). Moreover, miRNAs can act on animal behavior. It has been found that, in eukariotic organism (amphibian larvae), miRNAs participate in neuroplasticity (attraction/aversion) in relation to social preference to sustained exposure to kinship odorants. Thus, miRNAs act as a switch governing experience-dependent social preference (Dulcis et al., 2017).

MiRNA are capable of silencing RNA in a similar way to siRNA, but differing in terms of origins, as miRNA originate from self-folding regions of RNA transcripts forming short hairpins (Lim et al., 2003; Cuperus et al., 2011). Their action mode consists of an interaction with target mRNAs in a perfect complementary base sequence that results in mRNA cleaveage; furthermore, an interaction in an imperfect base sequence causes a translational repression (Yekta et al., 2004).

### Small interfering RNA (siRNA) or (RNAi)

Measure 20–25 bp, and originate from regions of double-stranded RNA molecules (dsRNA). These molecules are capable of interfering with mRNA translation by degrading it after transcription through perfect base pairing. *In vivo* and *in vitro* experiments suggest that the first RNAi initiating step involves the binding of the RNA nucleases to a large dsRNA and its cleavage into discrete 21- to 25-nucleotide RNA fragments (siRNA). In a second step, these siRNAs join a multinuclease complex (RISC) and degrade the homologous single-stranded mRNAs (Agrawal et al., 2003). SiRNA allows for the silencing of genes from different eukaryotic organisms with great specificity (Sunkar et al., 2007; Ghildiyal and Zamore, 2009). The specificity of siRNA's post-transcriptional gene silencing has been used in the development of therapeutic applications for treating a great variety of diseases (Zhou et al., 2008; Pulukuri et al., 2009).

### Small nuclear RNA (snRNA)

Are molecules around 150 bp in length. They are located in the nucleus of eukaryotic cells where they can be found mainly in the soluble fraction of the nucleoplasm, but also associated with the chromatin (Mondal et al., 2010). SnRNA control pre-messenger RNA and regulate the nuclear level of active positive transcription elongation factor b (P-TEFb), thus regulating RNA polymerase II (RNAPII) transcription in the nucleus (Muniz et al., 2013). Among snRNA, there are small nucleolar RNAs (snoRNAs) found in eukaryotic nucleolus and the Cajal bodies. These have several roles in ribosome synthesis, the regulation of alternative splicing, translation and oxidative stress. Moreover, both snRNA and snoRNAs are related to hereditary disorders and carcinogenesis (Mannoor et al., 2012).

### Piwi-interacting RNA (piRNA)

PiRNA are the most abundant and diverse ncRNA molecules found in animals, and have 26–31 bp lacking sequence conservation. They interact with encoding regulatory proteins piwi, configuring RNA-protein complexes associated with post-transcriptional gene silencing and epigenetic reprograming. In the germ line of several animal lineages, piRNA form the piRNA-induced silencing complex (piRISC), a configuration capable of silencing foreign transposable elements protecting genomic heredity integrity (Siomi et al., 2011). Moreover, piRNA play a critical role in genome rearrangement and transgenerational carriers of epigenetic information for genome programming (Ashe et al., 2012), affecting varied biological processes such as stem-cell functioning, tissue regeneration and pathogenic states such as cancer (Kim, 2006).

### Long ncRNA (lncRNA)

Are functionally diverse relatively long (more than 200 bp) regulatory ncRNA molecules (Kurokawa, 2015). Despite being the least-studied ncRNAs, so far, it has been demonstrated that lncRNA are capable of regulating themselves and that they function as transcriptional activators and post-transcriptional regulators in gene expression (Ponting et al., 2009). LncRNA controls protein regulator activity and separate them from their target DNA sequences. LncRNA operates as a scaffold platform for subcellular structures, regulating other ncRNAs. However, several lncRNA manufacture themselves in to small RNAs (Wilusz et al., 2009). For instance, some lncRNA are involved in the regulation of somatic tissue differentiation by associating directly with the protein and mRNA related to these processes (Kretz et al., 2012). Xist is an lncRNA that has an important role in the inactivation of one of the X chromosome in female mammals. X-inactivation is a process that equalizes gene expression between mammalian males and females.

## The role of ncRNAs in phenotypic variation

As a consequence of genome organization, the proteome of higher organisms is relatively conserved. For example, comparing humans and mice in terms of genetic coding for proteins, their structure is 99% similar (Mattick, 2001). Therefore, the principal mechanisms of phenotypic variation between species are located in the non-protein coding area of the genome. This suggests that ncRNAs have an important role contributing toward an explanation for the biological diversity in the evolution of species. Small RNAs receive or transmit information from and to the environment, which is stored in the epigenome (Mattick, 2001). The sequence of the small ncRNAs shows evolutionary conservation that in lncRNAs is smaller with certain exceptions (Louro et al., 2008; Guttman et al., 2009; Mercer et al., 2009).

Next we will refer to several mechanisms where the ncRNAs intervene, regulating genetic expression and generating new phenotypic variation such as: (1) DNA Methylation, Chromatin remodeling, and gene expression, (2) Epiallelic interaction, (3) RNA editing, (4) Splicing, (5) Genome imprinting, (6) Hox genes, homeotic mutations, and development, (7) Transgenerational epigenetic.

## NcRNAs and their role in DNA methylation, chromatin remodeling and gene expression

In eukaryotes, epigenetic mechanisms consist of DNA methylation or chromatin modification such as methylation or acetylation (Weigel and Colot, 2012). SiRNAs and lnc RNAs participate regulating gene expression by heterochromatinization (Richards and Elgin, 2002; Rangwala and Richards, 2004; Vella and Slack, 2005; Kim, 2006; Bird, 2007; Koerner et al., 2009; Luco and Misteli, 2011; Luco et al., 2011; Siomi et al., 2011; Chisholm et al., 2012). SiRNAs regulates DNA methylation in CpG dinucleotide in eukaryotes (Kawasaki and Taira, 2004; Klose and Bird, 2006; Suzuki and Bird, 2008; Lyko et al., 2010; Siegfried and Simon, 2010). Additionally, methylation gives extra regulation to those regions of DNA coding for proteins (Flanagan and Wild, 2007; Guttman et al., 2009; Rinn and Chang, 2012; Kulis et al., 2013; Sabin et al., 2013). Also siRNAs induce DNA histone H3 methylation in human cells (Kawasaki and Taira, 2004). Differential methylations during development are important in cell differentiation during the mitosis (Bird, 2002). LncRNAs intervene in methylation or in demethylation through interaction with various methyl transferase in cis or trans, directly or indirectly through a protein intermediate (Cao, 2014; Zhao et al., 2016).

In eukaryotes, miRNAs, piRNAs, and siRNAs also have a function in gene expression at the level of chromatin through histone methylation, acetylation, ubiquination, sumoylation, and phosphorylation. These epigenetic mechanisms regulate gene action in different parts of the chromosome and have an important role in heterochromatinization, replication, and transcription (Black, 2003; Bannister and Kouzarides, 2011; De Lucia and Dean, 2011; Keller and Bühler, 2013; Joh et al., 2014; Rivera et al., 2014).

## NcRNAs in epiallelic interaction and imprinting

In Mendelism, alleles remain unchanged and are thus transmitted to offspring. With epigenetics, it has been established that alleles can undergo modifications due to methylations, where ncRNAs can participate (Yan, 2014). Methylation of one of the alleles can change the expression of other alleles and produce an epimutation in a locus and originate an *epiallele* that is a group of otherwise identical genes that differ in the grade of methylation and originate novel phenotype that are heritable across generations (Rakyan et al., 2002; Yan, 2014). In *Arabidopsis thaliana* several epialleles related with siRNAs have been identified that correspond to different *Arabidopsis* ecotypes. These varieties present different gene expression characteristics, which are stably-maintained and transmitted to the offspring (Watson et al., 2014). The use of DNA methylation inhibitors can induce phenotypic variation in epialleles during meiosis, which can then be inherited and produce evolutionary change in the offspring (Weigel and Colot, 2012; House and Lukens, 2014; Ruden et al., 2015).

SiRNAS also explains an unusual allelic interaction where an allele in trans position modifies the expression of that allele, without altering their intimate nucleotide structure. These epigenetic interactions in a locus gave origin to the concepts of *paramutations* (Mahfouz, 2010, reviewed by Hollick, 2010). Furthermore, paramutation also extended the concept of *imprinting* and transgenerational heredity to a allelic interaction (Li et al., 1993). In imprinting, the epiallele has a different expression depending on whether it comes from the father or mother. A paradigm of this situation is what happens in the plant *A. thaliana*, where the MEA gene is only expressed in the phenotype of the endosperm, the maternal epiallele (Mahfouz, 2010). Moreover, paramutation has been described in maize by Brink in 1956 in a b1 locus that encodes for the pigment anthocyanin: the B′ allele of low expressivity that can cause changes in the allele B1 of high expressiveness. This change may be inherited for several generations. Both B′ and B1 have the same nucleotide sequence but differ in their methylation pattern (Coe, 1966; Brink et al., 1968; Hollick, 2010). Recently it has been discovered that siRNAs from a tandem repeat of non-coding DNA located in the b1 gene are involved in the paramutation in maize (Chandler, 2007).

In mice, the induced paramutation *white-tail-tip* has been reported using an insertional mutation in the Kit locus (Yuan et al., 2015). Microinjection into fertilized eggs of Kit-specific miRNAs induced a heritable white tail phenotype; however the specific mechanism of these miRNAs on chromatin remodeling is still unknown (Rassoulzadegan et al., 2006; Hollick, 2010). Maternal miRNAs and piRNAs seem to have an inhibitory effect on the germ line transmission of paramutations, meaning they are an important tool for understanding the mechanism of epigenetic transgenerational inheritance (Yuan et al., 2015).

## RNA editing and the ncRNAs, and their impact in the regulation of transcription

RNA editing is a special type of mutation in the primary nucleotide sequences in RNAs of eukaryotes, in the nucleus or in mitochondria where functionally different proteins are processed from a single gene. RNA editing was discovered in mitochondria of the protozoa *Trypanosome* where a special type of deletion or insertion of Uridine occurs (Benne et al., 1986; Feagin et al., 1988; Rubio et al., 2007). RNA editing not only occurs in the RNAs that participate in the protein synthesis, but also in ncRNAs such as miRNAs, siRNAs, and piRNAs (Gott and Emeson, 2000; Blanc and Davidson, 2003; Luciano et al., 2004; Liang and Landweber, 2007). Other similar edition of RNAs have been described, such as cytosine deamination and inosine by adenin substitution (Gommans et al., 2009). In higher eukaryotes, A to I RNA generates RNA and protein diversity, selectively reshaping coding and noncoding sequences in nuclear and mitochondria transcripts. The enzymes involved in this type of editing are adenosine deaminases (ADARs). The ADARs edit the duplex RNAs formed by ncRNAs, and can alter RNA functions, leading to an modified regulatory gene network of mRNAs and miRNAs and also siRNAs, piRNAs, and lncRNAs (Singh, 2013), A to I RNA editing may provide key links between neural development, nervous system function and neurological diseases. The ncRNAs and their alternative expression may alter the regulation of genetic machinery and to cause neurological diseases (Penn et al., 2013; Singh, 2013). The list of ncRNAs and their relation with RNA editing in brain development and disease in mammals is growing (Mehler and Mattick, 2007; Salta and De Strooper, 2012). Therefore, RNA editing could be one of the, previously underappreciated, driving forces for adaptive evolution (Gommans et al., 2009).

## NcRNAs and splicing

In 1977, Sharp and Roberts discovered RNA splicing, wherein genes are divided into exons and introns (Sharp, 2005). Thus, the structural genes are fractionated into introns that are spliced out from the precursor-messenger RNA (pre-mRNA) and in exons that are the expressed regions in mature mRNA (Berk and Sharp, 1977; Chow et al., 1977; Gilbert, 1978; Berk, 2016). Introns could self-cleave by acting as an enzyme (ribozymes). Now we know that there is alternative splicing and that specific genes produce different proteins, generating complex proteomes that explain the structural and functional complexity in the eukaryotes organism (Graveley, 2001; Black, 2003; Matlin et al., 2005; Pan et al., 2008; Wang et al., 2008; Nilsen and Graveley, 2010). Splicing from a pre-mRNA is an alternative mechanism for genetic regulation in higher eukaryotes. Variability in splicing model is an important source of protein diversity from the genetic code (Black, 2003).

In eukaryotes, the majority of pre-mRNAs are subject to alternative splicing, which can be regulated according to the developmental stage or cell type, or in response to signal transduction pathways (Black, 2003; Blencowe, 2006; House and Lynch, 2008). A large number of introns are sources of ncRNAs, such as mi RNAs, lncRNAs, piRNAS, and small circular RNAs, revealing the high complexity of the genomes and epigenome of eukaryotes (Tilgner et al., 2012; Yang, 2015). This evidence suggests these ncRNAs are involved in speciation processes (Lei et al., 2016). SnRNAs and proteins constituting spleciosoma, an enzyme that removes the introns, also participate in splicing (Wahl et al., 2009).

## NcRNAs and genomic imprinting

Genomic imprinting is an epigenetic transgenerational process that marks DNA in a sex-dependent manner, resulting in the differential expression of a gene depending on its parent of origin. Achieving an imprint requires establishing meiotically stable male and female imprints during gametogenesis and maintaining the imprinted state through DNA replication in the somatic cells of the embryo (MacDonald, 2012).

The term *imprinting* was taken from Konrad Lorenz who used it in the context of animal behavior. Helen Crouse (1960) used it in relation to dipterans of the Sciaridae family to explain the preferential removal of paternal X sex chromosomes in the somatic and germinative cells of the diptera of these sciarid flies (Crouse, 1960). During meiosis, sex X chromosomes acquire an imprint (mark) throughout the process in their passage toward the paternal line that determines a behavior opposite to that conferred by the maternal germ line (Crouse, 1960). Very similar phenomena, such as the heterochromatinization of paternal chromosomes occur in mealybug insects *Planococcus lilachinus* (Khosla et al., 1996; Bongiorni et al., 1999). For instance, in *P. citri* the haploid set of chromosomes of paternal origin, in males and females, is hypomethylated and heterochromatized, which does not happen with the haploid set derived from the mother (Brown and Nur, 1964; Brown, 1966; Bongiorni et al., 1999). Also, genomic imprinting has been found in mammals, demonstrating that androgenic and gynogenic zygotes were not functionally equivalent (McGrath and Solter, 1984; Feinberg, 2000).

Imprinting explains the inactivation by heterochromatinization of one of the sex X-chromosome in females of mammals, where one lncRNAs is transcripted from de Xist gene acting in cis position (Blignaut, 2012). The establishing of imprinting requires establishing epigenetic meiotically stable tags during meiosis in gametogenesis and also maintaining the imprinted state through DNA replication in the somatic and germinal cells of the embryo (MacDonald, 2012).

## NcRNAs and their relation with the hox gene, homeotic mutations and development

Homeotic mutations are reflected in drastic, often aberrant changes in an organism's phenotypic structures by another different during development (for example antennae by legs; Goldschmidt, 1945a,b; Dietrich, 2000). In Goldschmidt's opinion, these mutations are important in order to understand the developmental basis for morphological innovations and new species formation (Dietrich, 2003). However, these ideas were not taken into serious consideration the evolutionists of that time (Dobzhansky, 1940). Homeotic mutations are generally not adaptive, but some of them could pass the natural selection filter (Goldschmidt, 1940) and can explain the origin of biological novelties such as new species formation (Scott et al., 1989).

In light of current advances in epigenetic research, homeotic mutation could be a fundamental factor in organic evolution. In the last decade, it has been demonstrated that homeotic mutations that have to do with development in eukaryotes are controlled by ncRNAs (Petruk et al., 2006; Rinn et al., 2007). It has been discovered that miRNAs are encoded in homeotic genes (Hox genes). These miRNA genes are associated with transcription factor-encoding genes, and thus are of particular interest to the changes described above. In Hox genes there is a nucleotide sequence (homeo-domain) that is essential for embryonic development (McGinnis and Krumlauf, 1992). The homology between the homeotic invertebrated gene with vertebrate Hox genes has been demonstrated (Akam, 1989; Schubert et al., 1993; Fried et al., 2004). Therefore, these sequences are highly evolutionarily conserved and very important in the development of organism. The huge quantity of Hox miRNAs suggest that they play a significant role in Hox gene regulation during development through mRNA cleavage and translation inhibition (Yekta et al., 2004; Rinn et al., 2007).

Intergenic regions of the Hox genes in *Drosophila* produce many lncRNAs that regulate Hox gene coding sequences (Petruk et al., 2006). The studies of long ncRNAs have increased in recent times, and have become very important in expanding the knowledge of the regulation of development and other biological processes such as, heterochromatinization or diseases, and also in genomic changes (Kung et al., 2013).

## NcRNAs and transgenerational epigenetics

One of the great problems that Jacob and Monod solved was to find a mechanism of genetic regulation at the cellular level in *E. coli*, which they called *operon lactose* (Jacob and Monod, 1961, 1963). In the eukaryotes there were similar models that explained cellular differentiation and development (Gann, 2010). With the advances of molecular genetics, and the finding of several new modes of regulation of genetic action such as DNA methylation, histone modification and ncRNAS, the regulation of gene expression and cell differentiation has been better understood in eukaryotic organisms. These epigenetic changes, in differentiated somatic cells, can be transmitted during mitosis. But now we know that cell-to-cell inheritance can also be extended to meiotic generational inheritance between organisms (Tollefsbol, 2014). Traditionally, studies concerning the transfer of information between generations have focused on DNA as the only molecule that contains heritable genetic information, but now we know that in the epigenome there are also epigenetic marks that could be transgenerationally inherited (Jablonka et al., 2005). Epigenetic transgenerational inheritance has been defined as transmission via the germ line (sperm or egg) of epigenetic tags between generations in the absence of direct stimuli or genetic changes that drive phenotypic variation (Skinner, 2011; Yan, 2014; Yohn et al., 2015). Small ncRNAs are influential in transgenerational epigenetic inheritance because they can act as guides to specific genomic location by sequencing homology and also by recruiting various proteins to target sites, including epigenetic modifiers such as methyltransferases that are important in ADN methylation (Castel and Martienssen, 2013; Riddle, 2014).

In basal eukaryotes, such as *C. elegans*, transgenerational epigenetic inheritance mediated by ncRNAs has been described. The gene silencing induced by treatment with dsRNA in the parent is transgenerational, and inherited to the F1 offspring, proving that the silent state is transmitted through gametes to the next generation or past the F1 offspring where RNAi, siRNA, and piRNA pathways participate (Fire et al., 1998; Vastenhouw et al., 2006; Ashe et al., 2012; Riddle, 2014).

It has been found that in mammals there are various types of ncRNAs that can act in epigenetic programs. Epigenetic tags can be transmitted in somatic cells and also transgenerationally, where ncRNAs could correspond to a very important type of epigenetic inheritance mechanism (Larriba and del Mazo, 2016).

## Conclusion

In recent years, it has been demonstrated that ncRNAs participate in many important biological process in biodiversity that aren't included in classic evolutionary theory, such as phenotypic variation, regulation of gene expression, development and transgenerational epigenetic inheritance. With these new epigenetic mechanisms, several question arose in relation to the origin and maintenance of the biodiversity in populations. In this final section, we will then try to answer some of these questions.

## NcRNAs as interphase between the epigenotype and environment. genetic or epigenetic revolution?

Transposable elements, viruses and the RNA world, in particular the ncRNAs, open a new window into the knowledge of the processes explaining the dynamics of phenotypic changes, biodiversity and evolution. An increasing number of ncRNAS have been found in all life forms: from viruses and the simplest unicellular organisms (bacteria, archea) to the more complex eukaryotes such as mammals. These molecules have been revealed to have most varied functions, challenging the value C paradox, which was not really a paradox, but rather the lack of information regarding the functional values of an important and very dynamic area of an organism's inheritance: the epigenoma, where the different classes of ncRNAs play a fundamental role in generating evolutionary novelties.

NcRNAs participate in many biological processes, both in plants and animals, such as the regulation of transcription, development and adaptation to stressful conditions in the environment. In animals, lncRNAs regulate important processes in the central nervous system such as neurogenesis, neuron formation and synaptic plasticity related to behavior. With the advent of epigenetics and ncRNAs research, new sources of genetic variation and control of gene action have been discovered, such as splicing, RNA editing, metastable epialleles, and paramutations. NcRNAs actively participate in all these cases, generating dynamic responses to the environment and phenotypic novelties and giving rise to new species. Organisms can solve emerging problems that arise from the environment by increasing their epigenetic repertoire and dynamically developing distinct phenotypic variation without the need for new mutations or a *genetic revolution*, as has been postulated in the classic geographical model of speciation within the framework of the Synthetic Theory of Evolution (Mayr, 1949).

Furthermore, ncRNA molecules help to explain, from a molecular point of view, some classic concepts that are sources of phenotypic variation, such as pleiotropy and phenotypic plasticity. RNA splicing and RNA editing, although via different mechanisms, arise as updated explanations for the concept of pleiotropy, which itself is not adequately covered by Neo-Darwinian approaches. Plate, in 1910, describes the concept of pleitropy as a mutant gene with several phenotypic effects. As a consequence of splicing, one gene is capable of originating several proteins with different functions. This process has been proposed to be related with the increase diversity of proteomic and evolutionary diversification (Graveley, 2001; Bush et al., 2017). In RNA editing, epimutation at mRNA produces different versions of proteins with different functions in different cells (Gu et al., 2016). RNA editing increases the functional capacity of a single mRNA in different cells (Harjanto et al., 2016). This pleiotropic capacity of a unique mRNA to express itself in different cells and organs could develop varied organism phenotypes and responses in the face of environmental pressures in a rather adaptive fashion (Eddy, 2001; Mattick, 2001). In this new scenario, ncRNAs may become the artisans of the pleiotropic expression of a living organism's genome, allowing life on earth to thrive and colonize multiple habitats and overcome the boundaries of life (Khraiwesh et al., 2012; Wang et al., 2012). Considering this evidence, ncRNAs could be considered the precursor of speciation (Lake et al., 1988; Landweber and Gilbert, 1993). NcRNAs also provide an up-to-date heuristics tool for the consideration of the ontogenetic and phylogenetic consequences of environmentally inherited influences (Burggren et al., 2016).

Futhermore, it is probable that the evolution of new functional repeated RNAs has been derived from ncRNAs by retrotramposition. NcRNAs can diversify in their structure and adopt new roles (Herbert and Rich, 1999), extending the coding capacity of the genome to the epigenome. Thus, ncRNAs could be a reservoir for speciation and organic evolution (Matylla-Kulinska et al., 2014; Lei et al., 2016).

Splicing and RNA editing may also help to explain other classic concepts of phenotypic plasticity and the *norm of reaction* (Woltereck, 1909; Thoday, 1953); therefore, ncRNA appear to cover these previous definitions and processes with mechanisms.

## Mendelian or epigenetic inheritance?

Epigenetic variations in the epigenome would be inherited in a Neo-Lamarckian manner, bypassing the Weismann barrier and thus reviving Baldwin's old ideas (1896, 1897) of organic selection and Waddington's epigenetic heredity (2012) on genetic assimilation and inheritance produced by environmental pressures.

Now we know that phenotypic plasticity not only protects individuals from environmental changes, but also that there is an epigenetic control in these phenotypic changes (Moss, 2001), increasing the phenotypic variability at population level. In addition, new epigenetics tags in the epigenome could be transgenerationally inherited and populations of a species could have a different epigenetic mark but similar protein DNA code regions (Verhoeven et al., 2010; MacDonald, 2012). Experimental studies show that epigenetic variations, environmentally induced in phenotypic changes, could be inherited by future generations (Jablonka and Raz, 2009). Thus, the epigenetic variation in the epigenome corresponds to a new and important mechanism of phenotypic variation with an evolutionary perspective. This evidence has been collected from many species, including microorganisms (e.g., bacteria; Adam et al., 2008), plants (Hauser et al., 2011), and vertebrates. For instance, it has recently been described that populations of bats have different epigenetic marks suggesting that these epigenetic tags could have a correlation with phenotypic variation (Liu et al., 2015) and probably with speciation. In social insects, ncRNA related epigenetic changes have been found playing key roles in varied biological dynamics, from development to behavioral processes (Asgari, 2013). For example, studies of miRNA population diversity among *Apis mellifera* castes demonstrated striking differences between miRNA from nursing and foraging bees. Furthermore, in that study it was found that some of these ncRNA molecules were related to neural functions (Liu et al., 2012). Metastable epialleles and paramutations, which occur at the level of gene alleles, are also a source of novel epigenetic variability that help explain phenotypic variegation phenomena and also previously unknown aspects of classical quantitative genetics. These epigenetics changes would be inherited by genomic imprinting.

Environmental stressors induce epigenetic changes at epigenome level where several ncRNAs motile elements and viruses participate. These can explain some non-Mendelian models of inheredity. NcRNAs process and store a lot of information from environmental signals against unfavorable environmental conditions. In the adult rat it has been described that cells exposed to traumatic conditions during early life have different types and amounts of miRNAs in their blood, brain, and spermatozoids in comparison to the non-traumatized individuals. Some of these miRNAs were produced in excess while others were underrepresented in comparison with control animals. These changes resulted from deficient regulation of cell processes controlled by these miRNAs (Gapp et al., 2014). These behavioral symptoms were also observed in the offspring of treated groups, despite the fact that these pups were never exposed to stress during their own ontogeny, suggesting that germ line epigenetic marks were alerted due to the paternal stress and that such alteration was then inherited trough the spermatozoids (Gapp et al., 2014). It is becoming increasingly evident that the surrounding environment leaves epigenetic footprints on brains, organs, and also gametes, in which case epigenetic marks may even pass to the next generation (reviewed by Denhardt, 2017; Mulder et al., 2017). Thus, populations with their epigenetic repertoire increase the adaptive behavior and phenotypic plasticity of their individuals, allowing an organism's structural coupling with its environment (Maturana-Romesín and Mpodozis, 2000). All this is thanks to the development of distinct epigenotypes helped by ncRNAs and without concomitant mutations to the underlying genes. Under this novel epigenetic understanding of gene expression and phenotypic variation, we find an explanation for the current phenotypic variation and biodiversity on our planet, without resorting to mutation as the only source of evolution.

## Where does natural selection act?

Transgenerational epigenetic inheritance tells us that natural selection acts on the epigenome of the organism (Ruden et al., 2015), specifically on ncRNAs, which correspond to the interface between the genotype and the environment, capturing environmental signals. This contradicts one of the fundamental ideas of population genetics, which establishes that natural selection acts on the genotypes of the individuals in the population.

Making an analogy between an organism and a building: If a catastrophic event occurs, it acts directly on the building and not on the blueprints. The resistance of the building to the catastrophe will depend on the quality of the materials used in construction. The genome corresponds to the blueprints of the building, while the epigenome is the construction company and the workers who make the building (viruses, transposable elements, ncARNs). Biotic and abiotic environmental factors are fundamental during the development process, and that will depend on the capabilities that the organism has for overcoming the negative aspects of natural selection (Furrow, 2014; Burggren et al., 2016).

## How do new species originate? through mutations or through epimutations?

With the advent of epigenetics, and transgenerational inheritance, it is now possible to propose as a hypothesis that the very epigenetic mechanisms that regulate ontogenetic gene expression and cell differentiation also intervene in the origin of new species in a phylogenetic dimension. In other words, the organisms' behaviors in response to environmental pressures leave its epigenetic marks, via similar epigenetic paths (ncRNAs) both during individual's life as well as transgenerationally, through its progeny. NcRNAs are complementary to the role of proteins in the model proposed by Jacob and Monod, which refers to the mechanisms of regulation of gene expression during development (Gann, 2010). Both processes integrally contribute to an understanding of the mechanisms of organic development and evolution (EvoDevo) and the genome-epigenome circuit. For instance, the differences of structural genes in chimpanzees and humans is only about 4% (Varki and Altheide, 2005). However, the phenotypic differences between them are significantly higher and are probably due to differences in the epigenome of these species. Under current ncRNA evidence, speciation should be considered a process where the epigenomic changes are caused by the pressures of the environment. The landscape of ncRNAs in an organism not only allows cellular differentiation and development in eukaryotes, but also relief from the negative effects of stress and natural selection, as has been demonstrated in model system organisms as well as in our own species.

**Figure 1:**
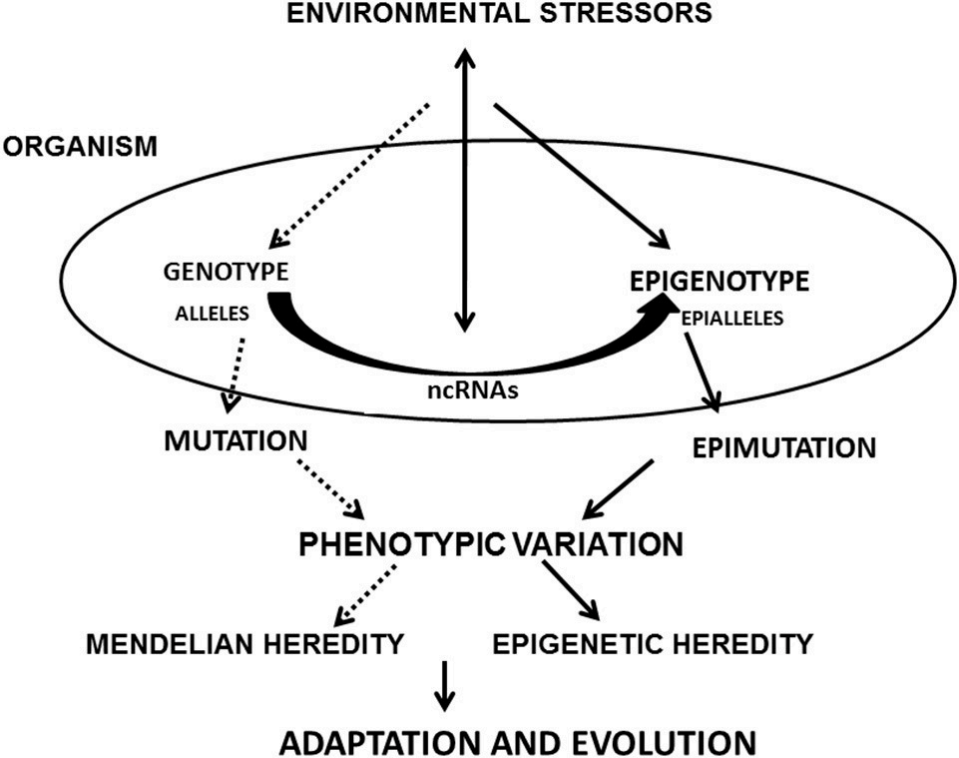
Flow Diagram showing the paths where ncRNAs are involved in the development of phentopic variations and evolution: environmental stressors act on the epigenotype and genotype. ncRNAs receive and respond to these stimuli. These epigenetic influences, together with the effect of mutations and Mendelian inheritance originate new adaptations and evolutionary noveties would arise.

Epigenetic changes involving ncRNAs that produce phenotype variability (epimutation, splicing, and RNA editing) may have an adaptive value for individuals who are carriers of these variations (Steele et al., 1998). However, they do not follow the Mendelian principles of heredity and are closer to the model proposed by Lamarck on the inheritance of acquired characteristics, foundations now denominated Neo-Lamarckism (Jablonka et al., 2005; Jablonka and Raz, 2009). Based on current findings, ncRNAs arise as active vehicles for epigenetic variation, phenotypic plasticity and heredity, revisiting classic concepts, and contributing with mechanistic explanatory power to a non-reductionist view of modern biology and the evolution of species (Figure 1).

## NcRNAs and their importance in molecular coadaptation and evolution

A genome's molecular structure, both in the animal and plant kingdom, demonstrates that ncRNAs are scattered among the species that constitute the three domains of the tree of life. These ncRNAs act as co-adapted endosymbiotic molecules with the genome and epigenome of their hosts and are the product of molecular coevolution from the origins of the first cells. With the exception of ribozyme, the most relictual molecules in organic evolution (as proposed by Gilbert, 1986), all the others ncRNAs require interaction with different protein molecules to exert their regulatory epigenetic function on genetic expression. Therefore, a primary stage in the evolutionary process that gave rise to the first cells, and the subsequent diversification of living forms, consisted of a molecular coevolution forming dynamic co-adapted molecular complexes. Without this molecular co-adaptation, organic evolution would not have been possible. The increasing number and diversity of these small and long ncRNAS in relation to the complexity and adaptability of living beings, explains that they have been paramount in complex biological processes and are not an evolutionary paradox.

The fact that miRNAs can be mobilized by the fluids of plants and animals, allows them to act at different distances to where they were transcribed, much like hormones or pheromones do. In addition, they can respond to environmental stimuli, favoring the adaptation of organisms through the modification of epigenetic marks and also a transgenerational inheredity and the evolution of species as part of a Neo-Lamarckian model.

## Author contributions

Main hypothesis developed by DF-L. Blibliographic review and secondary writing by CV.

### Conflict of interest statement

The authors declare that the research was conducted in the absence of any commercial or financial relationships that could be construed as a potential conflict of interest.
